# Disrupting heroin-associated memory reconsolidation through actin polymerization inhibition in the nucleus accumbens core

**DOI:** 10.1093/ijnp/pyae065

**Published:** 2024-12-24

**Authors:** Haiting Zhao, Haoyu Li, Li Meng, Peng Du, Xin Mo, Mengqi Gong, Jiaxin Chen, Yiwei Liao

**Affiliations:** National Clinical Research Center for Geriatric Disorders, Xiangya Hospital, Central South University, Changsha, China; Department of Neurology, Xiangya Hospital, Central South University, Changsha, Hunan, China; National Clinical Research Center for Geriatric Disorders, Xiangya Hospital, Central South University, Changsha, China; Department of Neurosurgery, Xiangya Hospital, Central South University, Changsha, Hunan, China; National Clinical Research Center for Geriatric Disorders, Xiangya Hospital, Central South University, Changsha, China; Department of Radiology, Xiangya Hospital, Central South University, Changsha, Hunan, China; Department of Neurosurgery, The Second Affiliated Hospital, Xinjiang Medical University, Urumqi, China; National Clinical Research Center for Geriatric Disorders, Xiangya Hospital, Central South University, Changsha, China; Department of Neurosurgery, Xiangya Hospital, Central South University, Changsha, Hunan, China; National Clinical Research Center for Geriatric Disorders, Xiangya Hospital, Central South University, Changsha, China; Department of Neurosurgery, Xiangya Hospital, Central South University, Changsha, Hunan, China; National Clinical Research Center for Geriatric Disorders, Xiangya Hospital, Central South University, Changsha, China; Department of Neurology, Xiangya Hospital, Central South University, Changsha, Hunan, China; National Clinical Research Center for Geriatric Disorders, Xiangya Hospital, Central South University, Changsha, China; Department of Neurosurgery, Xiangya Hospital, Central South University, Changsha, Hunan, China

**Keywords:** heroin, actin polymerization, reconsolidation, nucleus accumbens core, rats

## Abstract

**Background:**

Understanding drug addiction as a disorder of maladaptive learning, where drug-associated or environmental cues trigger drug cravings and seeking, is crucial for developing effective treatments. Actin polymerization, a biochemical process, plays a crucial role in drug-related memory formation, particularly evident in conditioned place preference paradigms involving drugs like morphine and methamphetamine. However, the role of actin polymerization in the reconsolidation of heroin-associated memories remains understudied.

**Methods:**

This study employed a rodent model of self-administered heroin to investigate the involvement of actin polymerization in the reconsolidation of heroin-associated memories. Rats underwent ten days of intravenous heroin self-administration paired with conditioned cues. Subsequently, a 10-day extinction phase aimed to reduce heroin-seeking behaviors. Following this, rats participated in a 15-minute retrieval trial with or without cues. Immediately post-retrieval, rats received bilateral injections of the actin polymerization inhibitor Latrunculin A (Lat A) into the nucleus accumbens core (NACc), a critical brain region for memory reconsolidation.

**Results:**

Immediate administration of Lat A into the NACc post-retrieval significantly reduced cue-induced and heroin-primed reinstatement of heroin-seeking behavior for at least 28 days. However, administering Lat A 6-hour post-retrieval or without a retrieval trial, as well as administering Jasplakionlide prior to memory reactivation did not affect heroin-seeking behaviors.

**Conclusions:**

Inhibiting actin polymerization during the reconsolidation window disrupts heroin-associated memory reconsolidation, leading to decreased heroin-seeking behavior and prevention of relapse. These effects are contingent upon the presence of a retrieval trial and exhibit temporal specificity, shedding light on addiction mechanisms and potential therapeutic interventions.

**Significance Statement**

Drug addiction is often viewed as maladaptive learning, where drug-related cues trigger cravings and drive drug-seeking behavior. Actin polymerization plays a crucial role in drug-related memory formation, especially in paradigms like conditioned place preference with morphine and methamphetamine. This study investigated the impact of actin polymerization on heroin-associated memories using a rodent model of self-administered heroin. Rats underwent heroin training paired with light and tone cues, followed by extinction and a retrieval trial. Bilateral injection of the actin polymerization inhibitor Latrunculin A into the nucleus accumbens core immediately post-retrieval significantly reduced cue-induced and heroin-primed reinstatement for 28 days. This study demonstrates that disrupting actin polymerization during reconsolidation reduces heroin-seeking behavior, highlighting temporal specificity in therapeutic interventions against relapse. These findings offer valuable insights into addiction mechanisms and underscore the critical timing of interventions to effectively combat relapse.

## INTRODUCTION

Substance addiction is a chronic brain disorder marked by a high propensity for relapse. Individuals with this disorder often experience intense cravings long after withdrawal, leading to high relapse rates.^1-3^ Despite being aware of the severe consequences, these individuals continue to engage in compulsive drug-seeking and drug-taking behaviors.^4–7^ Therefore, a critical issue in addressing substance addiction treatment is the prevention of relapse post-detoxification. The extensive impact of addiction reaches beyond the personal challenges faced by individuals, inflicting significant economic and social costs on society. As per the World Drug Report 2022, over 38.6 million people were estimated to be affected by drug use disorders.^8^ The crisis is evident not only through the direct physiological impact of the substances but also via a complex network of associative memories that underpin addiction.

Drug-related memories, which are resistant to eradication, play a central role in maintaining compulsive drug behaviors and relapse after withdrawal.^9-12^ The formation of conditioned associations between drug use and environmental cues is key to developing addiction behaviors.^13-15^ Understanding the neural mechanisms of associative learning during the formation of these memories is crucial for developing new relapse-prevention strategies based on memory and learning theories.

A key concept in comprehending and potentially disrupting these memories is “reconsolidation.”^16^ Memories become unstable and malleable during a 6-hour window upon recall,^17-19^ offering a strategic opportunity to intervene and potentially disrupt drug-seeking behaviors.^15,20,21^ By focusing on reconsolidation, researchers and clinicians can potentially weaken or alter these addictive memories each time they are reactivated, offering a unique opportunity to disrupt the cycle of addiction outside the narrow consolidation window.^15,22^ Disruption of reconsolidation in drug-related memory models has been shown to effectively reduce drug-seeking behaviors and prevent relapse.^18,23^ Hence, targeting reconsolidation of drug-related memories emerges as a promising therapeutic approach for the treatment of drug addiction and relapse prevention.

Although the exact mechanisms behind drug memory reconsolidation are not fully understood, evidence is mounting that disruptions in actin dynamics could be critically involved in this process.^24-26^ Actin serves as a fundamental structural element of cells, crucial for preserving cell shape and functionality.^27^ The dynamic rearrangement of actin is of paramount importance in various memory process.^28,29^ For instance, the stabilization of actin filaments is associated with the consolidation of synaptic strengthening.^30^ Moreover, studies indicate that the actin filament dynamics within the amygdala disrupt both the consolidation and reconsolidation of fear-related memories.^24,31^ Furthermore, alterations in actin cytoskeletal dynamics are also implicated in encoding of drug-associated memories, including morphine,^24,32^ methamphetamine,^26,33-36^ and alcohol.^37,38^ Collectively, these lines of evidence suggest that actin dynamics may be critically involved in the re-stabilization of heroin-associated memories.

The nucleus accumbens (NAC) plays a crucial role in the brain’s reward circuitry and is integral to the development of drug addiction.^39,40^ This region consists of 2 main subregions: the core and the shell. The core primarily triggers motor responses and drug-seeking behaviors, while the shell is involved in the emotional aspects of drug consumption and reinforces drug use.^41-45^ Chronic exposure to opioids like heroin significantly affects the dendritic spine density and synaptic structure within the NAC, though the effects are varied and complex. Studies suggest that early opioid exposure alters synaptic connectivity, which may enhance compulsive drug-seeking behavior. Over time, changes in spine density may either increase or decrease, indicating a bidirectional effect potentially driven by the drug’s influence on different signaling pathways in the brain’s reward system.^46^ Research highlights the role of drebrin, an actin-binding protein, in spine morphology and synaptic stability, which is crucial in the neuroadaptive processes linked to prolonged opioid use.^47^ Given these insights, targeting the molecular pathways involved in synaptic architecture presents a promising therapeutic avenue. For instance, interventions that inhibit actin polymerization could theoretically weaken the drug-related synaptic connections, thereby reducing the likelihood of relapse. Nonetheless, the exact roles and mechanisms by which the NACc influences the reconsolidation of heroin-associated memories are still under investigation.

In current research, it has been noted that inhibiting actin polymerization in the NAC core (NACc) can prevent heroin relapse by disrupting the reconsolidation of heroin-associated memories. This inhibition remains effective for more than 28 days following a single dose of the actin polymerization inhibitor. Importantly, administering the inhibitor outside the reconsolidation time window does not affect the heroin-associated memory. These findings suggest that targeting actin polymerization during memory reconsolidation could be an innovative approach to combating drug relapse.

## MATERIALS AND METHODS

### Subjects

Adult male Sprague–Dawley rats, weighing between 280 and 300 g, were housed under a 12:12 light/dark cycle, with food and water ad libitum. All procedures were performed in accordance with the National Research Council’s Guide for the Care and Use of Laboratory Animals. All animals were handled 3 minutes for 5 days before behavioral conditioning. In this experiment, a cohort of 72 rats was utilized, with the exclusion of 6 due to unsuccessful acquisition of self-administered heroin or issues with catheter patency. Specifically, we defined stable responding as having variability in the number of self-administered injections within a 20% range across the last 3 consecutive days.

### Surgery

We anesthetized the rats using isoflurane (1%–2% for maintenance) and inserted a thin flexible tube into the left jugular vein near the left atrium. The catheter was fastened to the vein using a silk suture and then routed the external segment of the catheter to an exit port located on the back of the neck, then under the skin to the skull’s apex, where it was affixed to a modified 22-gauge cannula. This cannula was secured onto the skull using screws and dental cement, ensuring a stable and durable connection. This setup allowed for free movement within the behavioral box while ensuring secure and reliable drug delivery during self-administration sessions. Rats to recover for 7 days after surgery. 23-gauge guide cannulas (Plastics One) were implanted 1 mm above the NACc. The specified stereotaxic coordinates for NACc were as follows: anteroposterior, +1.5 mm; mediolateral, ±3.8 mm; and dorsoventral, −6.2 mm from the skull surface.^48-50^

### Drugs

**Figure 1.**
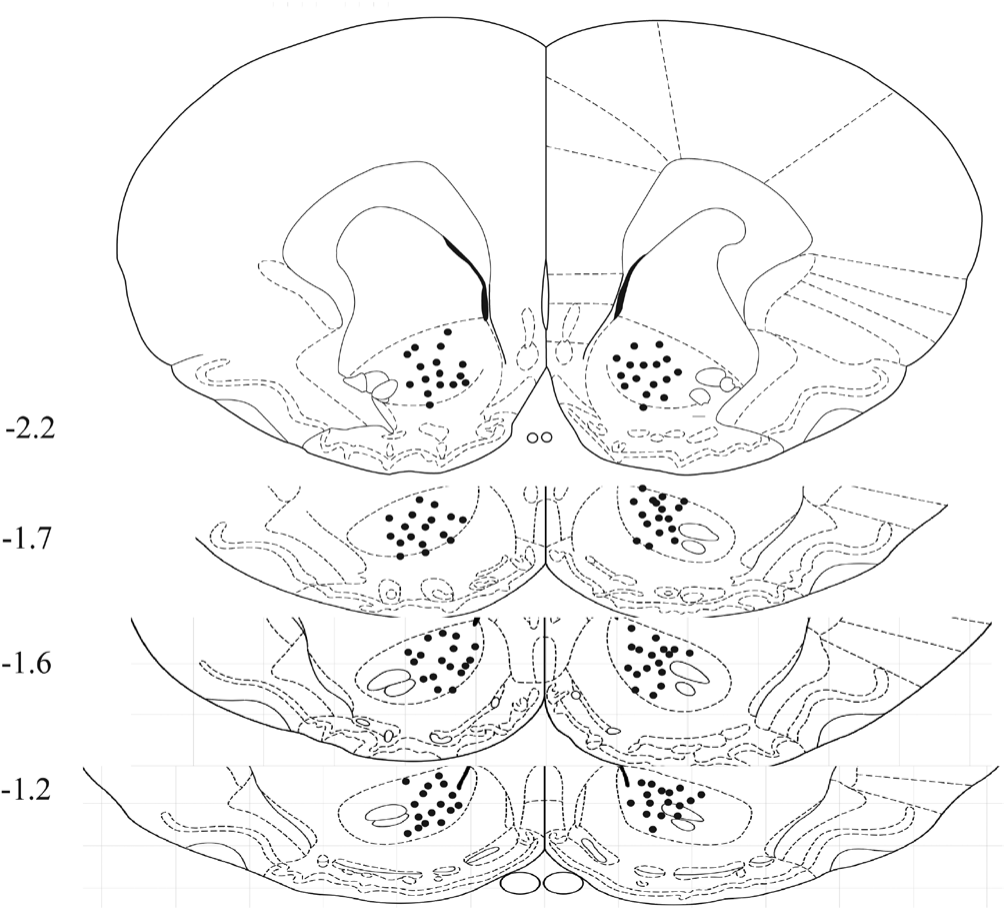
Schematic representation of injection sites. A diagram displays the locations of infusions, with numbers indicating their positions relative to bregma (mm) along the anterior-posterior axis.

Latrunculin A (Lat A), an actin polymerization inhibitor, was obtained from Abcam Jasplakinolide (Jas), an inducer of actin polymerization was purchased from Invitrogen. For experimental administration, Lat A or Jas were initially dissolved in DMSO to create a stock solution with a concentration of 25 μg/μL. Then, this stock solution was further diluted in PBS until reaching a final concentration of 0.5 μg/μL, and either this preparation or an equivalent volume of vehicle (0.5 μL) was administered bilaterally into the NACc with or without memory retrieval. The selected dosage for Lat A was determined in alignment with previous studies.^32,51^ All infusion sites in the NACc are shown in Figure 1.

### Intravenous Heroin Self-administration Training

The testing chambers, provided by AniLab Software & Instruments, included 2 nosepoke apparatuses positioned 5 cm from the chamber’s base. Interactions with the left apparatus (deemed active) resulted in heroin doses, paired with a 5-second auditory–visual cue. Although actions with the right apparatus (considered inactive) were recorded, they did not yield any outcome. Over a span of 10 days, rats were conditioned to acquire intravenous heroin dosages of 0.05 mg/kg per infusion during 3 daily hour-long intervals, each separated by a 5-minute gap. These sessions coincided with the beginning of their nocturnal cycle. We employed an FR1 reinforcement paradigm, introducing a 40-second pause post each infusion. Every session was initiated with a house light activation, which was sustained throughout.

### Nosepoke Extinction

During the extinction sessions (180 minutes), the experimental setup replicated the conditions consistent with the heroin self-administration training; however, the modification for this phase entailed solely recording the number of nosepokes without heroin infusions or presenting the associated tone-light cue. During this, the rats extinguish the contextual and the operant response.

### Cue Extinction

During the extinction sessions (180 minutes), the experimental setup replicated the conditions consistent with the heroin self-administration training; however, the extinction session took place in a different context (context B, experiment 5).

### Memory Retrieval

In a 15-minute memory retrieval session, the rats’ active nosepoke actions triggered the conditional presentation of a 5-second auditory and visual cue, without the reinforcement of heroin delivery.

### Cue Exposure Extinction

During the cue exposure extinction sessions (180 minutes), the experimental setup replicated the conditions consistent with the heroin self-administration training; however, the modification for this phase entailed the rats’ active nosepoke actions triggered the conditional presentation of a 5-second auditory and visual cue, but not heroin infusions (experiments 1, 3, and 4).

### Test for Context-, Cue-, and Drug-Induced Reinstatement

In both 1-hour context-, cue-, and drug-induced reinstatement test, the experimental setup replicated the conditions consistent with the heroin self-administration training; however, the modification for this phase entailed the rats’ active nosepoke actions triggered the conditional presentation of a 5-second auditory and visual cue, but not heroin infusions. Specifically, in the drug-induced reinstatement test, rats were injected heroin (0.25 mg/kg, subcutaneously) 5 minutes before re-entering the test context.

### Spontaneous Recovery Test

During the spontaneous recovery test, the experimental setup replicated the conditions consistent with the cue-induced reinstatement test. The number of nosepokes were recorded for 1 hour after 28 days of withdrawal.

### Experiments Design

Experiment 1: Effect of bidirectional manipulation of actin before or after reactivation of the heroin-associated memory on subsequent reinstatement of heroin-seeking behavior.

Experiment 1 evaluated whether intra-NACc infusion of Lat A or Jas, which inhibits or induces the actin polymerization, respectively, would impair instrumental heroin-associated memory reconsolidation. After 10-day acquisition and 10-day nosepoke extinction sessions, the rats were re-exposed to the heroin-paired context to initiate the destabilization and reconsolidation of heroin-associated memories. Immediately after or before the session, rats received bilateral intra-NACc microinfusions of Jas (0.5 μL) or Lat A (0.5 μL). Subsequently, a cue-induced relapse test was carried out 1 day after the manipulation. Following the test, rats received an additional 2-day cue extinction. Twenty-four hours later, a drug-priming test was performed immediately after administration of heroin.

Experiment 2: Effect of intra-NACc infusion of Lat A immediately after reactivation of the heroin-associated memory on spontaneous recovery of heroin-seeking behavior.

Experiment 2 assessed whether microinfusions with Lat A in NACc have a long-term effect on heroin-seeking behavior. After a stable acquisition and extinction training, rats underwent a 15-mintue retrieval followed by microinfusion with Lat A into the NACc. A cue-induced relapse test was carried out 24 houtd after the manipulation. Finally, on day 55, 1-hour spontaneous recovery test for heroin relapse was conducted after 28 days.

Experiment 3: Effect of intra-NACc infusion of Lat A without reactivation of the heroin-associated memory on subsequent reinstatement of heroin-seeking behavior. Experiment 3 examined whether the effects of intra-NACc Lat A administration would depend on explicit memory reactivation. The experimental protocol was the same as Experiment 1 except that no memory retrieval session before intra-NACc infusion of Lat A.

Experiment 4: Effect of intra-NACc infusion of Lat A 6 hours after reactivation of the heroin-associated memory on subsequent reinstatement of heroin-seeking behavior. Experiment 4 investigated whether a 6-hour-delayed intra-NACc Lat A infusion after memory reactivation would disrupt heroin-associated memory. Aside from the 6-hour delay in administering intra-NACc Lat A, the experimental procedure was identical to that of Experiment 1.

Experiment 5: Effect of intra-NACc infusion of Lat A immediately after reactivation of the heroin-associated memory on subsequent contextual heroin-associated memories.

Experiment 5 further investigated intra-NACc infusion of LatA after cue-induced reactivation also disrupted contextual heroin-associated memories. Aside from the different contexts during the extinction session, the experimental procedure was identical to that of Experiment 1.

### Statistical Analyses

No statistical methods were used to predetermine sample sizes, but the sample sizes used are similar to those generally employed in the field. All behavioral tests and analyses were blindly conducted. We used GraphPad v.8.0 to analyze the data, presented as mean ± SEM. Repeated-measure ANOVAs were used with a between-subjects of different administration (Lat A or control) factors and a within-subjects of different test factors, followed by Bonferroni’s post hoc test. Values of *P* < .05 were considered to be statistically significant.

## RESULTS

1. Effect of bidirectional manipulation of actin before or after reactivation of the heroin-associated memory on subsequent cue- and drug-induced heroin-seeking behavior.

**Figure 2.**
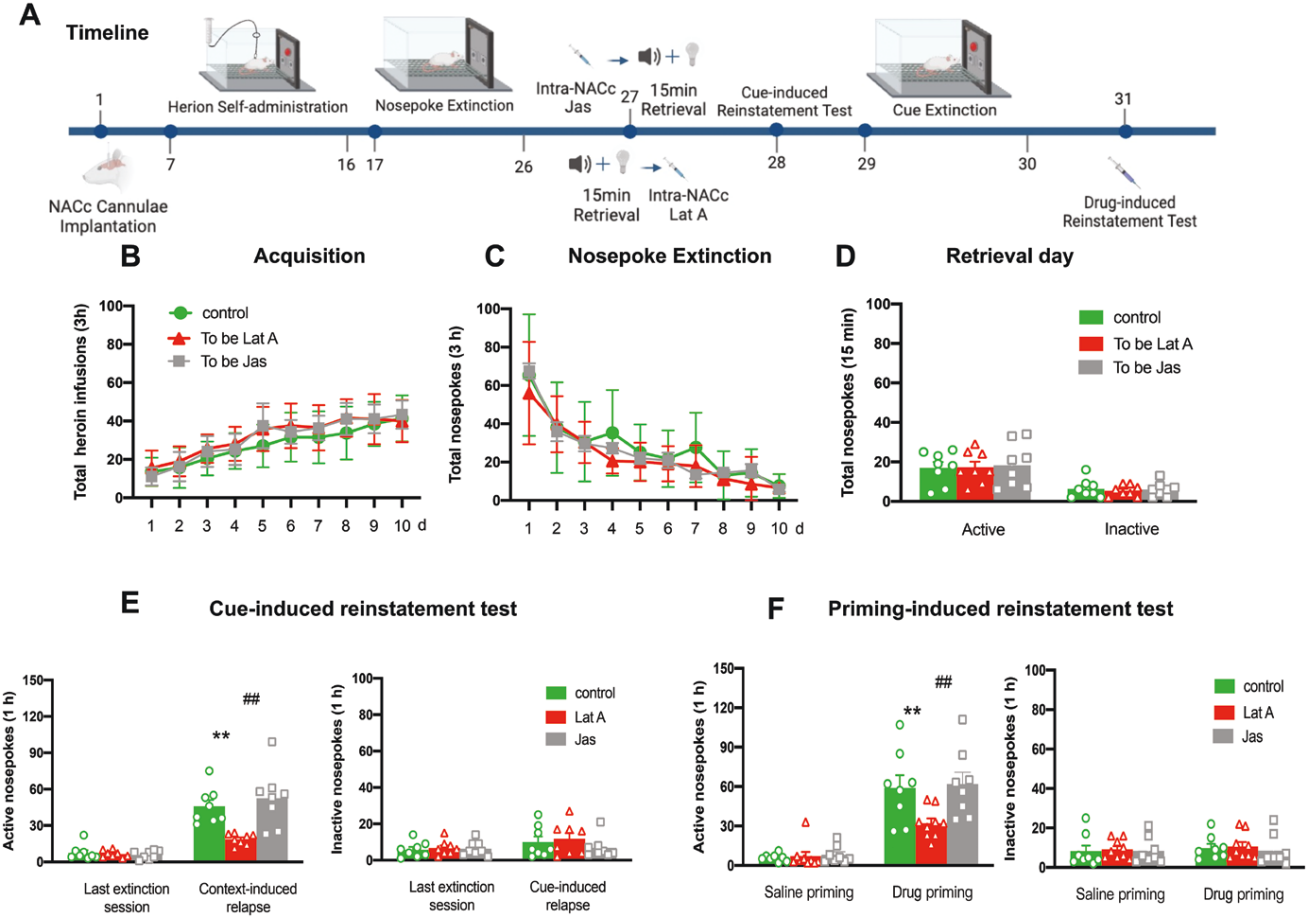
Intra-NACc infusion of Lat A during the reconsolidation process inhibits the expression of relapse induced by cue- or heroin-priming. (A) Schematic of experimental design. (B) The mean number of heroin infusion during the acquisition phase. (C) The mean number of active nosepoke during the extinction phase. (D) The mean number of active and inactive nosepoke during the retrieval session. (E) The mean number of active (Left column) and inactive (Right column) nosepoke during the last extinction session and cue-induced reinstatement test. (F) The mean number of active (Left column) and inactive (Right column) nosepoke during the saline priming and drug-priming reinstatement test. Control *n* = 8; To be Lat A/Lat A *n* = 9; To be Jas/Jas *n* = 8. (**,^##^) *P *< .01. Error bars represent SEM.

To examine whether actin polymerization is essential for the reconsolidation of heroin-associated memories. Three groups of rats were trained for 10-day heroin self-administration and 10 consecutive days of nosepoke extinction. On day 27, immediately after or before the memory retrieval, rats received bilateral intra-NACc microinfusions of Jas (*n* = 8), Lat A (*n* = 9), or Control (*n* = 8). The cue- and drug-induced reinstatement tests were performed on days 28 and 33, respectively. During this, a 2-day cue extinction session was conducted (Figure 2A). Statistical analysis revealed that no group differences for total heroin infusions number (*F* [18, 189] = 0.875, *P* = .609 for Group × Time interaction) during acquisition training (Figure 2B), total nosepoke number (*F* [18, 189] = 0.814, *P* = .682 for Group × Time interaction) during extinction training (Figure 2C), as well as the number of active nosepoke (*P* > .1) and inactive nosepoke (*P* > .1) in memory retrieval (Figure 2D). In addition, the intra-NACc infusion of Lat A decreased the number of active nosepoke in both cue- (*F* [2, 22] = 6.97, *P* = .004 for Group × Time interaction) and drug-induced (*F* [2, 22] = 3.861, *P* = .03 for Group × Time interaction) heroin-seeking behavior, but did not alter the number of inactive nosepoke (*P* > .1). Furthermore, the Jas group has no significant differences in the number of active nosepoke in both cue- and drug-seeking behavior, as well as the number of inactive nosepoke, compared to the control group (All *P* > .1) (Figure 2E and F). These findings suggest that microinjection of Lat A, but not Jas into the NACc during the reconsolidation process can inhibit the expression of relapse induced by cue- or heroin-priming.

2. Intra-NACc infusion of Lat A immediately following memory retrieval has long-lasting effects in preventing heroin relapse.

**Figure 3.**
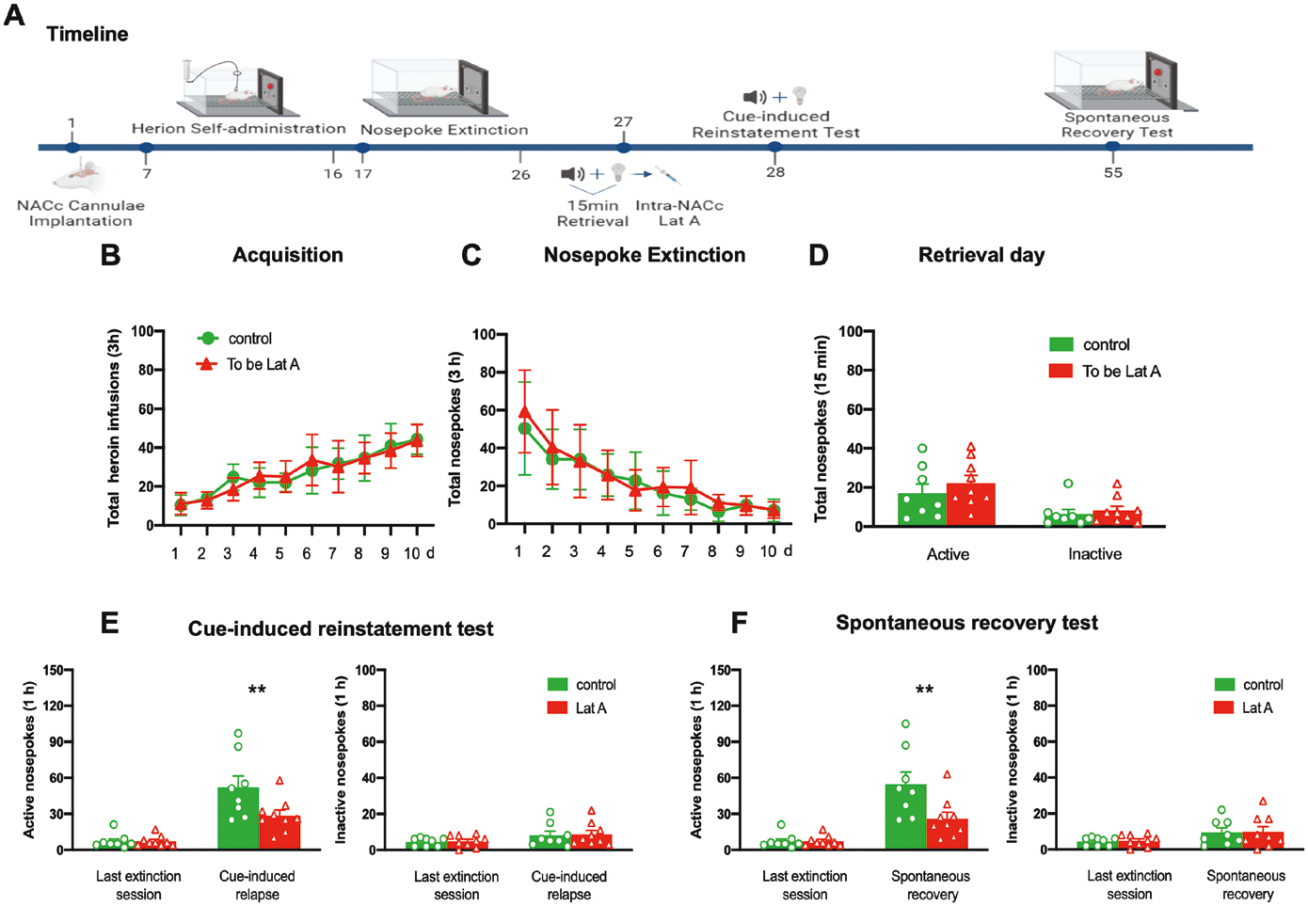
Intra-NACc infusion of Lat A immediately following memory retrieval has long-lasting effects in preventing heroin relapse. (A) Schematic of experimental design. (B) The mean number of heroin infusion during the acquisition phase. (C) The mean number of active nosepoke during the extinction phase. (D) The mean number of active and inactive nosepoke during the retrieval session. (E) The mean number of active (Left column) and inactive (Right column) nosepoke during the last extinction session and cue-induced reinstatement test. (F) The mean number of active (Left column) and inactive (Right column) nosepoke during the last extinction session and spontaneous recovery test. Control *n* = 8; To be Lat A/Lat A *n* = 9. (**) *P *< .01. Error bars represent SEM.

To observe the long-term effect of Lat A on the reconsolidation of heroin-associated memory, rats were tested for a relapse of heroin-seeking behavior 28 days after the cue-induced reinstatement test (Figure 3A). No group differences for total heroin infusions number (*F* [9, 135] = 1.014, *P* = .432 for Group × Time interaction) during acquisition training (Figure 3B), total nosepoke number (*F* [9, 135] = 0.5138, *P* = .863 for Group × Time interaction) during extinction training (Figure 3C), as well as the number of active nosepoke (*P* > .1) and inactive nosepoke (*P* > .1) in memory retrieval (Figure 3D). In addition, the intra-NACc infusion of Lat A decreased the number of active nosepoke in cue-induced reinstatement of heroin-seeking behavior (*F* [1, 15] = 5.304, *P* = .036 for Group × Time interaction) (Figure 3E) and heroin-seeking behavior did not recover in the groups of rats administered Lat A (*F* [1, 15] = 7.379, *P* = .016 for Group × Time interaction) (Figure 3F). There were no significant differences in the number of inactive nosepoke in the cue-induced reinstatement test or spontaneous recovery test (*P* > .1) (Figure 3E and F). These findings suggest that the inhibition effect of Lat A on the heroin relapse lasts at least 28 days.

3. Administration of Lat A into the NACc without memory retrieval failed to alter drug-induced heroin-seeking behavior.

**Figure 4.**
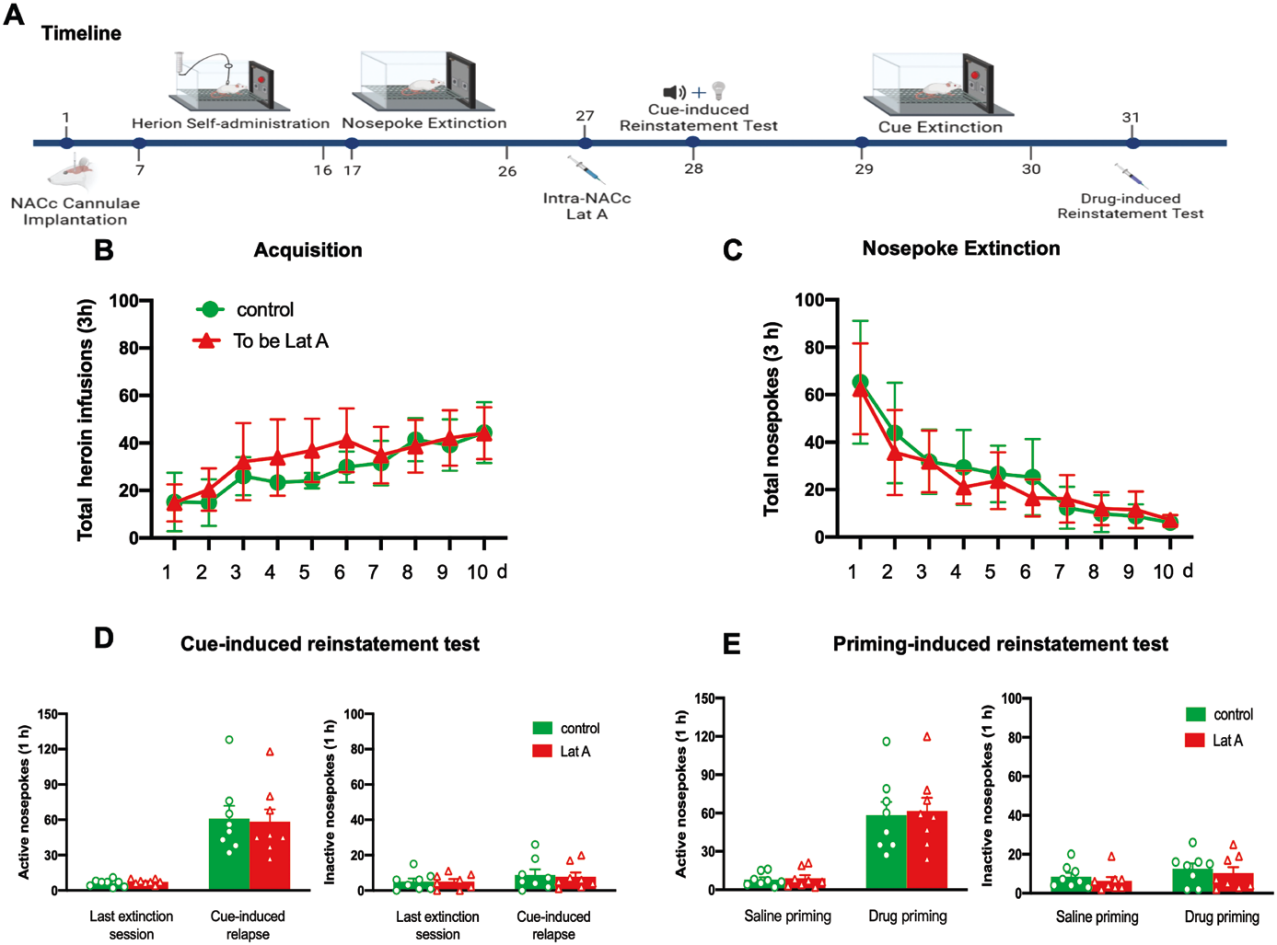
Administration of Lat A into the NACc without memory retrieval failed to alter drug-induced heroin-seeking behavior. (A) Schematic of experimental design. (B) The mean number of heroin infusion during the acquisition phase. (C) The mean number of active nosepoke during the extinction phase. (D) The mean number of active (Left column) and inactive (Right column) nosepoke during the last extinction session and cue-induced reinstatement test. (E) The mean number of active (Left column) and inactive (Right column) nosepoke during the saline priming and drug-priming reinstatement test. Control *n* = 8; To be Lat A/Lat A *n* = 8. Error bars represent SEM.

Next, we further investigated whether the inhibition effect of Lat A on heroin-seeking behavior is memory retrieval dependent. The experiment procedure was identical to experiment 1, expert that no retrieval session performed before intra-NACc infusion of Lat A (Figure 4A). No significant differences were found for total heroin infusion number (*F* [9, 126] = 1.510, *P* = .151 for Group × Time interaction) during acquisition training (Figure 4B) and total nosepoke number (*F* [9, 126] = 0.8185, *P* = .6 for Group × Time interaction) during extinction training (Figure 4C). In addition, no significant group differences were found for both active and inactive nosepokes in cue- and drug-induced reinstatement tests (all *P* > .1). These results suggest that the administration of Lat A into the NACc without subsequent memory retrieval fails to alter drug-induced heroin-seeking behavior.

4. Intra-NACc Lat A administration outside the reconsolidation window does not affect heroin-seeking behavior.

**Figure 5.**
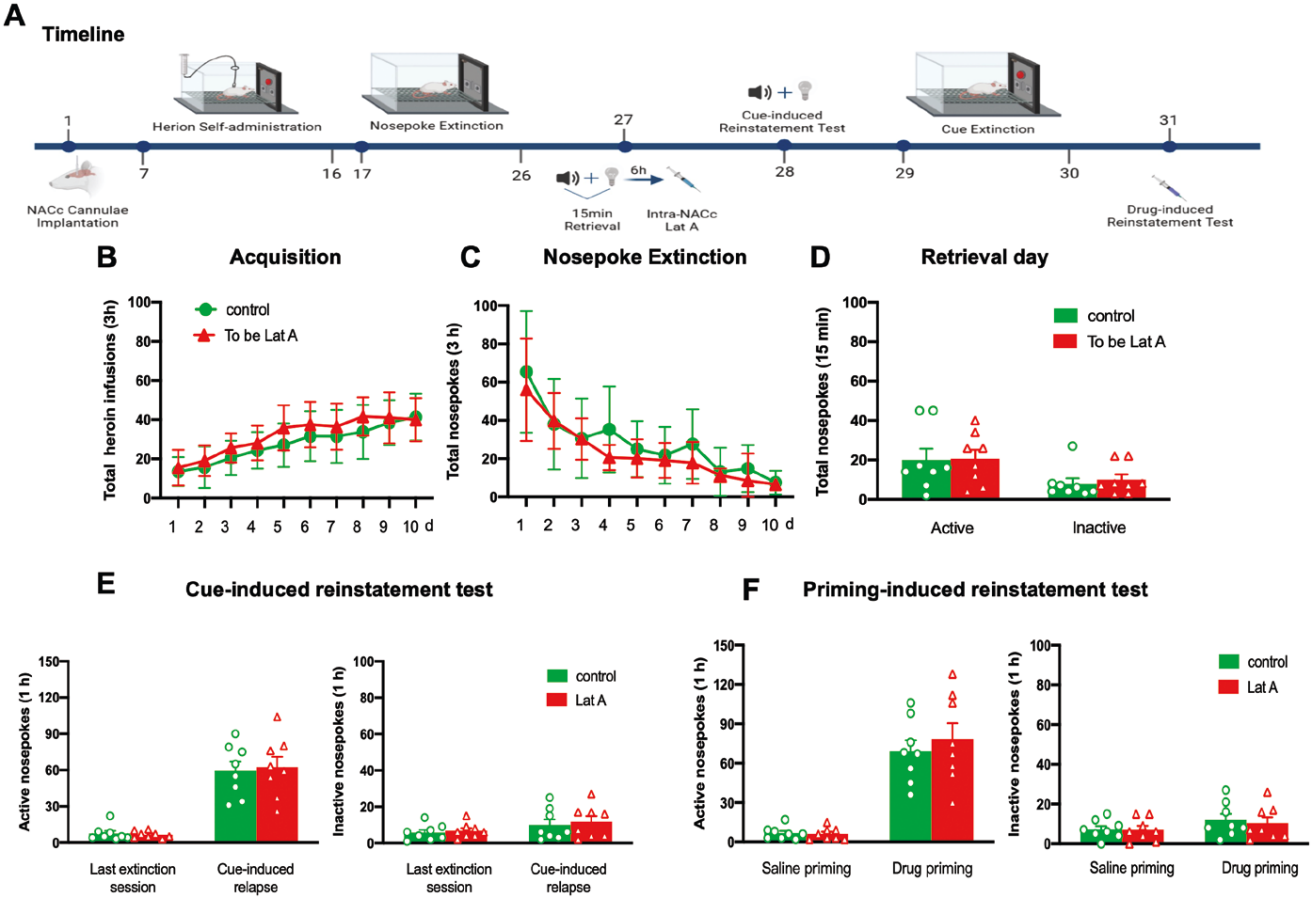
Intra-NACc Lat A administration outside the reconsolidation window does not affect heroin-seeking behavior. (A) Schematic of experimental design. (B) The mean number of heroin infusion during the acquisition phase. (C) The mean number of active nosepoke during the extinction phase. (D) The mean number of active and inactive nosepoke during the retrieval session. (E) The mean number of active (Left column) and inactive (Right column) nosepoke during the last extinction session and cue-induced reinstatement test. (F) The mean number of active (Left column) and inactive (Right column) nosepoke during the saline priming and drug-priming reinstatement test. Control *n* = 8; To be Lat A/Lat A *n* = 8. Error bars represent SEM.

As mentioned in the Introduction, the memory reconsolidation process continued for 6 hours. Hence, we tested the effect of intra-NACc of Lat A outside the time window on subsequent heroin-seeking behavior. Statistical analysis revealed that no groups differences for total heroin infusions number (*F* [9, 126] = 0.8347, *P* = .586 for Group × Time interaction) during acquisition training (Figure 5B), total nosepoke number (*F* [9, 126] = 0.7119, *P* = .697 for Group × Time interaction) during extinction training (Figure 5C), as well as the number of active nosepoke (*P* > .1) and inactive nosepoke (*P* > .1) in memory retrieval (Figure 5D). In addition, no significant group differences were found for both active and inactive nosepokes in cue- and drug-induced reinstatement tests (All *P* > .1) (Figure 5D and E). These results indicate that the effect of Lat A on the reconsolidation of heroin-associated memory is temporally specific. The findings demonstrate a time-dependent impact of Lat A on the reconsolidation of heroin-associated memories.

5. Intra-NACc infusion of Lat A during the reconsolidation process inhibits the expression of relapse induced by context.

**Figure 6.**
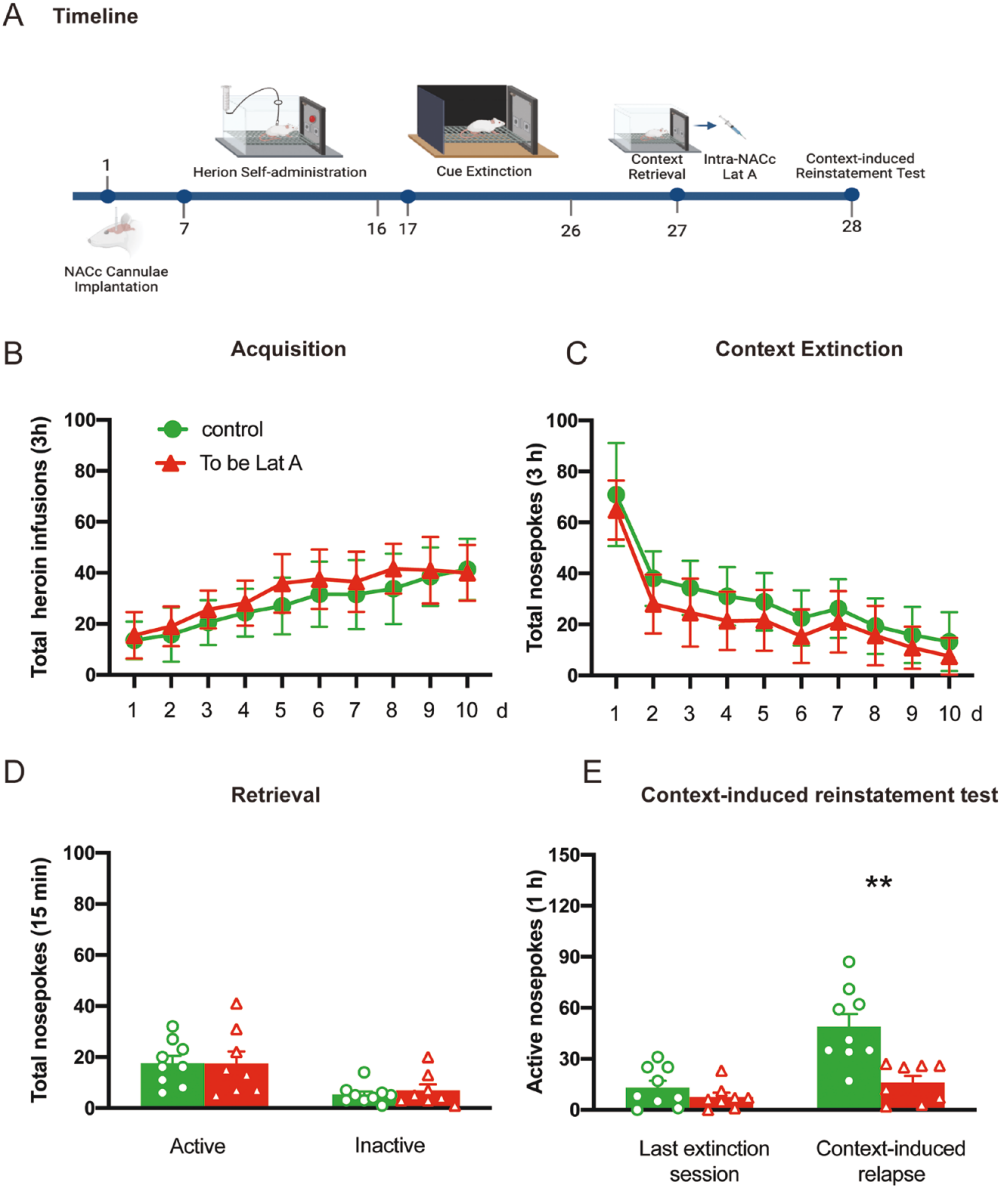
Intra-NACc infusion of Lat A during the reconsolidation process inhibits the expression of relapse induced by context. (A) Schematic of experimental design. (B) The mean number of heroin infusion during the acquisition phase. (C) The mean number of active nosepoke during the extinction phase. (D) The mean number of active and inactive nosepoke during the retrieval session. (E) The mean number of active nosepoke during the last extinction session and context-induced reinstatement test. Control *n* = 8; To be Lat A/Lat A *n* = 8. (**) *P *< .01. Error bars represent SEM.

Besides cue-associated memories, we also investigated whether the inhibition effect of Lat A could affect contextual heroin-associated memories. Two groups of rats were trained for 10-day heroin self-administration and 10 consecutive days of context extinction. On day 27, immediately after the memory retrieval, rats received bilateral intra-NACc microinfusions of Lat A (*n* = 8) or Control (*n* = 9). The context-induced reinstatement test was performed on day 28. Statistical analysis revealed that no groups differences for total heroin infusions number (*F* [9, 135] = 0.7268, *P* = .684 for Group × Time interaction) during acquisition training (Figure 6B), total nosepoke number (*F* [9, 135] = 0.3265, *P* = .965 for Group × Time interaction) during extinction training (Figure 6C), as well as the number of active nosepoke (*P* > .1) and inactive nosepoke (*P* > .1) in memory retrieval (Figure 6D). In addition, no significant group differences were found for active nosepokes in the context-induced reinstatement test (*P* > .1) (Figure 6E). These results indicate that the effect of Lat A on the reconsolidation of heroin-associated memory can also inhibit the expression of relapse induced by context.

## DISCUSSION

This study provides the first confirmation of the role of actin polymerization inhibition in the reconsolidation of heroin-associated memories via a classical self-administration model in rats. Previous research has demonstrated that actin dynamics are integral to memory consolidation and reconsolidation.^52-55^ Additionally, actin rearrangement is essential for the formation of drug-associated memories. The intricate process of actin rearrangement facilitates the structural changes in synapses that underpin the encoding and retrieval of memories associated with drug use. These synaptic modifications are crucial for the persistent and powerful nature of drug-related memories. While studies have suggested that actin polymerization is involved in drug-associated memories, direct evidence of its effect on heroin-associated instrumental behavior memories was lacking.

In this study, we adhered to the traditional experimental protocol to investigate the reconsolidation of addictive memories through a self-administration paradigm. After establishing a stable rat heroin self-administration model, we conducted an extinction procedure involving nose poking as a repetitive behavior. During the nosepoking extinction process, drug-associated cues were not presented, only the behavior of nosepoking was extinguished. In the memory retrieval trial, rats were re-exposed to a 15-minute session in the training chamber, during which nosepoking was accompanied by drug-associated cue presentation.^56,57^ Post-memory retrieval, the immediate microinjection of Lat A into the NACc disrupted actin polymerization. This disruption was found to suppress the reinitiation of drug-seeking behavior triggered by drug-associated cues on the following day, and heroin relapse did not reemerge during the heroin-priming test or under conditions of spontaneous recovery. Conversely, the application of Lat A 6 hours post-retrieval or in the absence of memory retrieval did not affect drug-seeking behavior elicited by drug-related cues or heroin-priming. These results indicate that microinjection of Lat A into the NACc disrupts reconsolidation of heroin-associated memories, instead of merely suppressing the expression of these memories. Prior research indicates that memory reconsolidation is a process dependent on new protein synthesis and typically occurs within a 6-hour time frame following retrieval.^58-62^ The success of strategies designed to interfere with memory reconsolidation hinges on the precise timing of the intervention as well as the activation of memory retrieval. Our results are consistent with the attributes of memory reconsolidation processes, as the disruption of actin polymerization in the NACc is dependent on memory retrieval and exhibits a clear temporal specificity. Therefore, actin polymerization in the NACc is implicated in the reconsolidation of heroin-associated memory.

Actin polymerization refers to the cellular process of assembling actin filaments, which is crucial in various drug-related memory processes. Modulation actin dynamics has been shown to facilitate the development of alcohol preference,^37^ and the manipulating intracellular actin regulators, such as dominant negative Rac or activated cofilin in the NAC can result in CPP even with a subthreshold level of cocaine.^63^ Lat A, the drug utilized in our research, binds to G-actin monomers and inhibits the polymerization process required to form actin filaments.^64^ However, Fujiwara et al. noted that Lat A can drive actin filament depolymerization at lower concentrations,^65^ but at higher concentrations in our study, its primary effect remains the inhibition of actin polymerization by sequestering G-actin monomers.^66-68^ In our study, we performed bidirectional manipulation of actin during the reconsolidation of drug-related memories. We found that inhibiting actin polymerization with Lat A, but not stabilizing actin with Jas, reduced both cue- and drug-induced seeking behavior in rats, suggesting that actin polymerization plays a critical role in the relapse of heroin-seeking behavior. Notably, enhancing polymerization did not affect seeking behavior; however, there is still a possibility that depolymerization could lead to memory deficits, which warrants further investigation.

Research findings have shown that administering Lat A into the BLA post-training disrupts the consolidation of methamphetamine-induced CPP.^26^ Furthermore, injecting Lat A into the NAC shell impairs the consolidation of morphine-induced CPP, suggesting a role for actin polymerization in the memory processes associated with drug addiction.^32^ In our work, we found that post-retrieval intra-NACc injection of Lat A disrupts the reconsolidation of heroin-associated memories and has a long effect on preventing relapse. Yet, the role of Lat A in drug memory appears inconsistent. Young et al.’s findings suggest that actin polymerization in the BLA only affects the consolidation of methamphetamine-induced CPP, but does not affect the reconsolidation process. Furthermore, Li et al.’s research indicates that only actin polymerization in the NAC shell erases morphine-induced CPP via disrupt reconsolidation but not the NACc. These discrepancies underscore the complexity of memory processes in drug addiction and suggest that the impact of interventions like actin polymerization depends on the specific brain region targeted and the addictive drug involved. First, different animal models to mimic drug abuse in humans (self-administration vs. CPP). The CPP model primarily reflects conditioned preferences in response to drug-associated cues, while the SA model more closely simulates voluntary and active drug-seeking behavior, resembling aspects of human relapse.^69-71^ Notably, the CPP model primarily involves brain regions related to learning and memory, such as the hippocampus and amygdala, while the SA model focuses on brain regions associated with reward and voluntary drug seeking, including the hypothalamus and NAC.^44,72^ Second, the NACc, NAC shell, and BLA exhibit distinct functional involvements in the mechanisms governing memory, potentially leading to variable outcomes of Lat A on the processes of memory acquisition, retention, and persistence.^73-75^ Specifically, the BLA predominantly participates in the emotional modulation of memory processes, whereas the NAC is principally implicated in the retention of memories associated with drug-induced rewards.^75-79^ Methamphetamine and heroin both exhibit notable prevalence within the sphere of illicit substance use, but they induce addiction through distinct mechanisms, which might underlie their differential effects on actin polymerization inhibition. Specifically, methamphetamine induces a surge in dopamine concentrations by obstructing the reuptake of dopamine via the dopamine transporter and by enhancing the release of dopamine.^80-82^ On the other hand, heroin, a derivative of morphine, induces dopamine release indirectly through the activation of opioid receptors, leading to a cascade of neurochemical events that culminate in dopamine secretion.^83,84^

Research has shown that actin’s dynamic state influences synaptic plasticity, which is pivotal for memory reconsolidation processes. The depolymerization of actin filaments disrupts the structural integrity of synapses, which is necessary for the modification of existing memories upon reactivation. This mechanism is especially relevant in the context of drug addiction, where memories associated with drug cues are often strong and persistent. By destabilizing these memories through actin depolymerization, it may be possible to weaken the associations between drug cues and the addictive behaviors they trigger. Thus, a deeper investigation into actin cycling—both polymerization and depolymerization—across various brain regions and in response to different drugs is crucial. Such studies would benefit from exploring not only the biochemical impacts of agents like Lat A but also the timing of their application in relation to memory reactivation. This could lead to more nuanced strategies that target specific phases of memory processing, offering a way to selectively impair unwanted drug memories without interfering with other cognitive functions.

We acknowledge several limitations of the present study. A notable limitation of this study we only focus on behavioral tests, excluding experiments in genomics and metabolomics. Future research should address this gap by exploring the molecular mechanisms underlying Lat A’s impact on the reconsolidation of heroin-associated memory. Another limitation is that we only used male animals. Considering that gender is a critical biological factor that influences the development of drug addiction,^85,86^ future studies should replicate these findings with female subjects is essential for broader generalization. Notably, the reconsolidation window, during which memories become malleable, is relatively brief. Achieving precise timing in drug delivery to maximize therapeutic effects translate to clinic remains a challenge. Finally, our animal research suggests that the NACc is a potential brain region for drug interventions. However, the administration of a specific drug to a particular brain region remains a challenge. With the development of viral vectors, gene therapy enables the delivery of biologics (DNA, mRNA, and CRISPR genome editors) to the entire brain or specific brain regions.^87-89^ Although still in its infancy, this strategy offers an optimistic path for the clinical translation of drug intervention experiments.

In summary, we found that inhibiting actin polymerization in the NACc during the reconsolidation process plays a critical role in the relapse of heroin-seeking behavior. Our research offers a novel perspective, indicating that inhibiting actin polymerization holds promise as a strategy for preventing relapse. Furthermore, our findings suggest that the timing of this intervention is critical and should occur within a precise period after retrieval to be effective. In future studiesy, it is interesting to use advanced neuroimaging techniques to visualize how actin polymerization affects neural networks in real time could provide deeper mechanistic insights.

## Data Availability

The data generated during this study are made available from the corresponding author upon reasonable request.
